# Supplementary material for: Colorimetric identification of colorless acid vapors using a metal-organic framework-based sensor
Source: Nat Commun. 2025 Jan 4;16:385. doi: 10.1038/s41467-024-55774-x (PMC11700211; doi:10.1038/s41467-024-55774-x)
Supplement: Supplementary file 1 — Supplementary information [file 41467_2024_55774_MOESM1_ESM.pdf]

Supplementary Information

Colorimetric Identification of Colorless Acid Vapors using a Metal-Organic Framework-Based Sensor

Wonhyeong Jang[†], Hyejin Yoo[†], Dongjun Shin, Seokjin Noh, and Jin Yeong Kim*

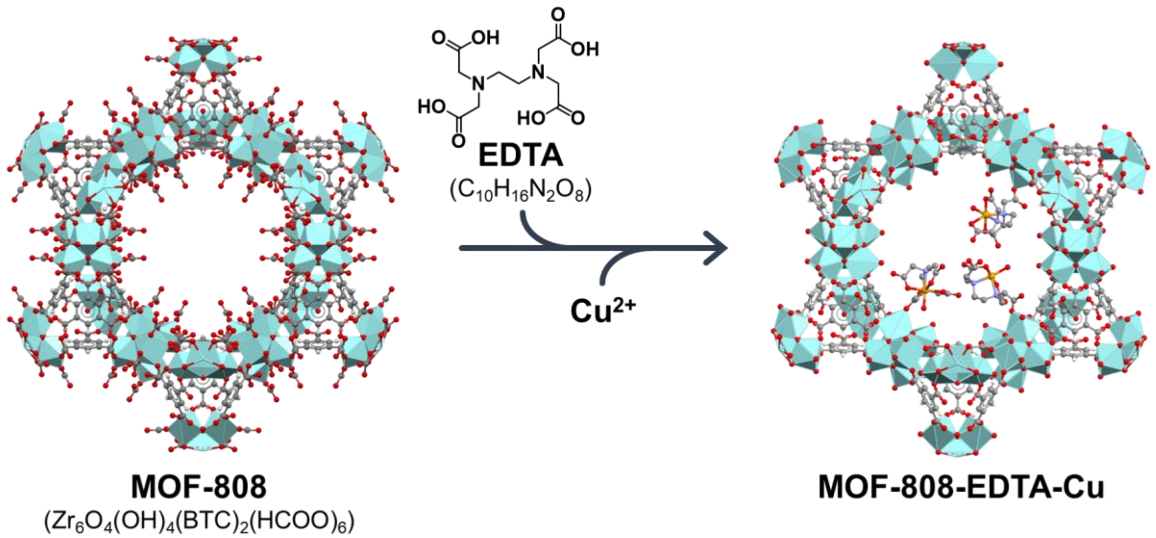

Supplementary Figure 1. Synthesis scheme of MOF-808-EDTA-Cu. BTC is an abbreviation for 1,3,5-benzenetricarboxylic acid. Zirconium (light blue), carbon (gray), oxygen (red), nitrogen (violet), and copper (orange).

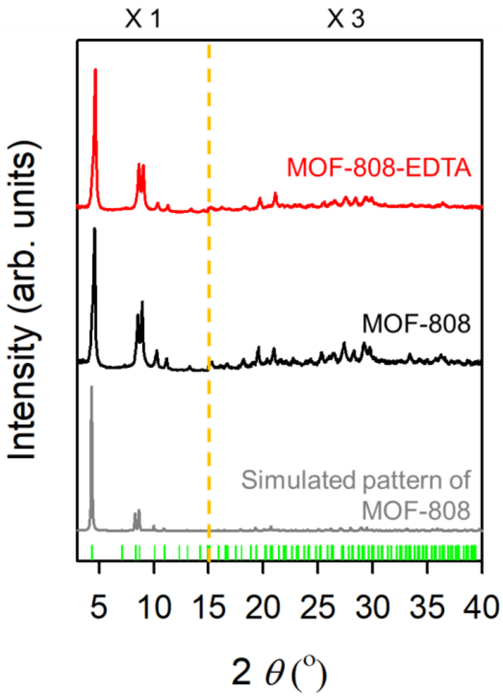

Supplementary Figure 2. XRPD patterns of MOF-808 (black) and MOF-808-EDTA (red) with simulated XRPD pattern of MOF-808 (gray). The intensity was tripled for the range of 2θ from 15 to 40 degrees, with and orange dash line marking 15 degrees.

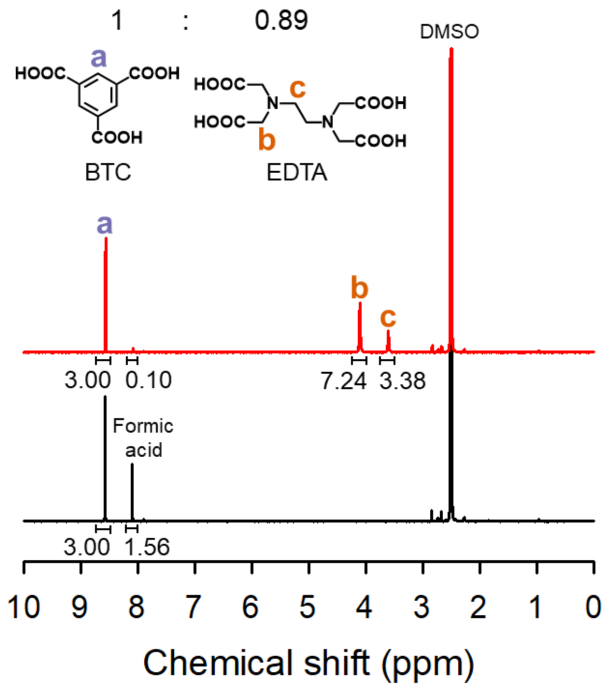

Supplementary Figure 3. ^{1}H NMR spectra of MOF-808 (black) and MOF-808-EDTA (red) in D_2SO_4/DMSO-d_6 solution. Based on the ^{1}H NMR spectrum of MOF-808-EDTA, the mole ratio of EDTA to 1,3,5-benzenetricarboxylic acid (BTC) is 0.89. Note that formic acid originates from MOF-808, which has the ideal structural formula $[Zr_6O_4(OH)_4(BTC)_2(HCOO)_6]$.

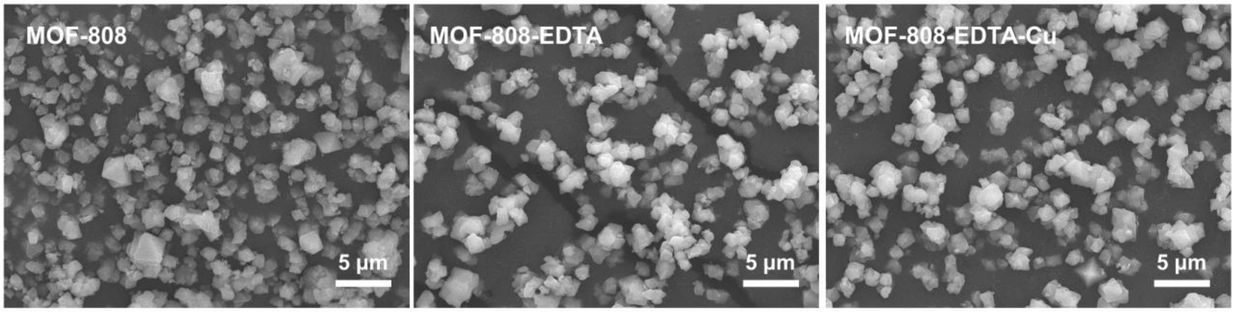

Supplementary Figure 4. SEM images of MOF-808, MOF-808EDTA and MOF-808-EDTA-Cu.

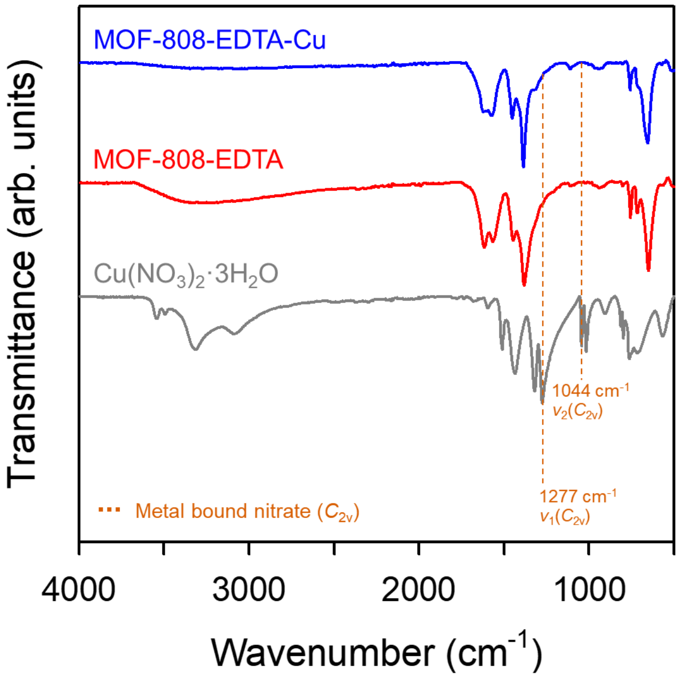

Supplementary Figure 5. FT-IR spectra of MOF-808-EDTA-Cu (blue), MOF-808-EDTA (red), and $Cu(NO_3)_2{\cdot}3H_2O$ (gray).

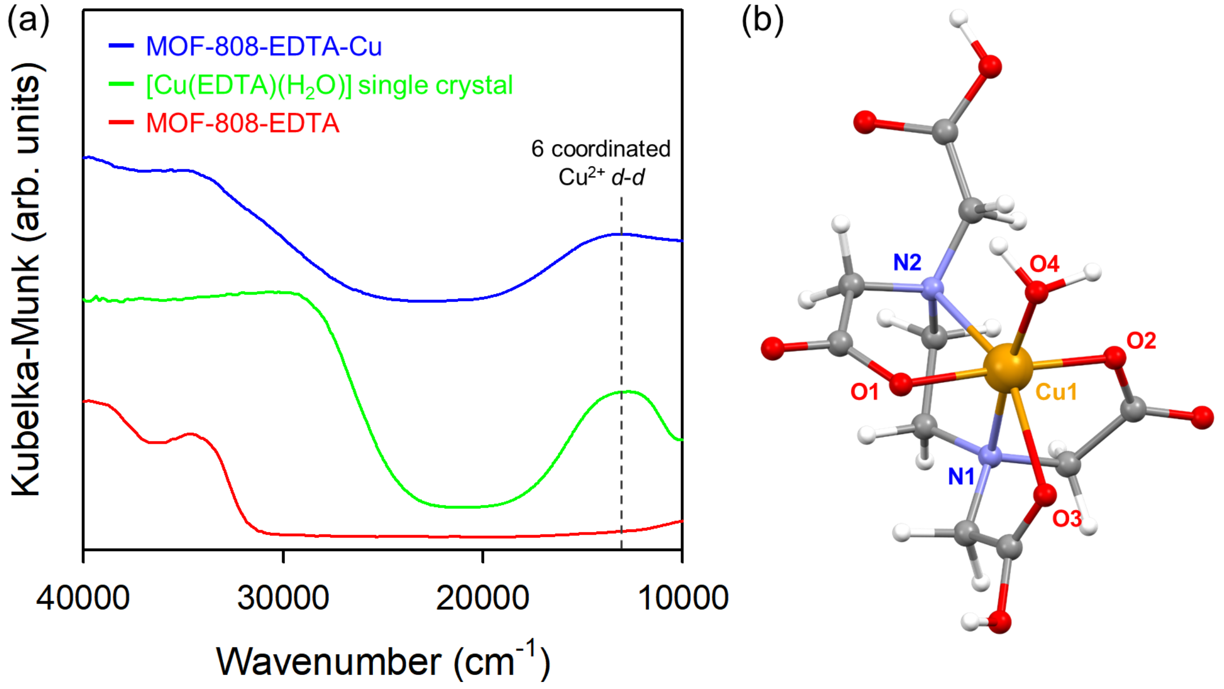

Supplementary Figure 6. (a) Diffuse reflectance UV-vis-NIR spectra of MOF-808-EDTA-Cu (blue), [Cu(EDTA)(H_2O)] single crystal (green), and MOF-808-EDTA (red). For MOF-808-EDTA-Cu (blue) and [Cu(EDTA)(H_2O)] single crystal (green), the peak appeared at ca. 13140 cm^{-1} is corresponding to *d*-*d* transition of an octahedral six-coordinated of Cu ion. (b) Crystal structure of [Cu(EDTA)(H_2O)], i.e., [Cu($C_{10}H_{14}N_2O_8$)(H_2O)], with six-coordinated geometry of Cu as a ball-and-stick model[1]. Carbon (gray), oxygen (red), nitrogen (violet), and copper (orange)

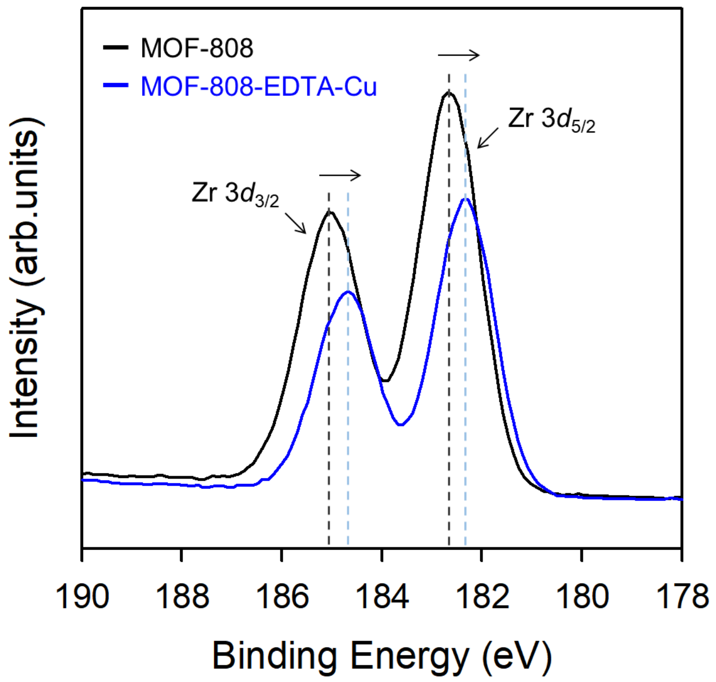

Supplementary Figure 7. Zr 3*d* XPS spectra of MOF-808 (black) and MOF-808-EDTA-Cu (blue).

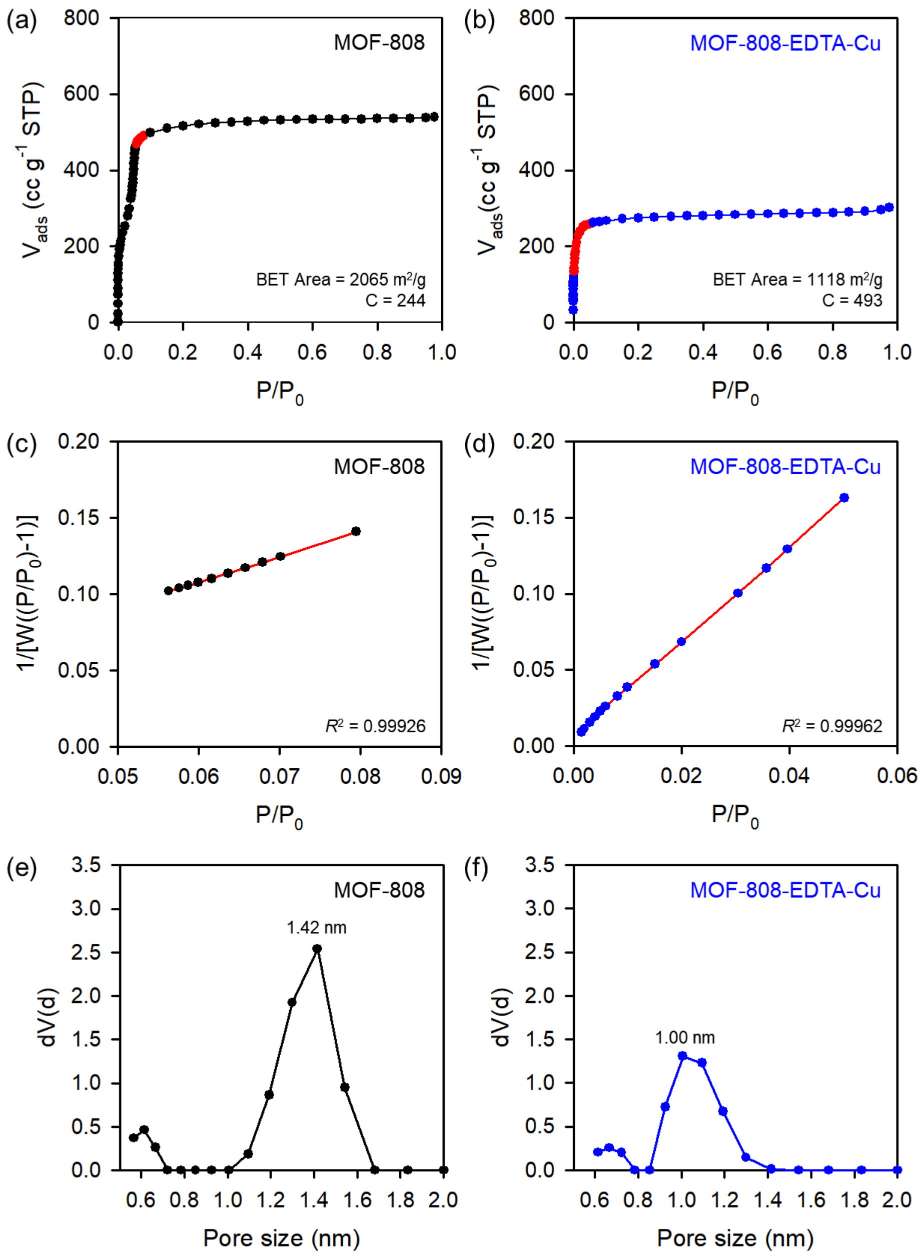

Supplementary Figure 8. N_2 adsorption isotherms of (a) MOF-808 and (b) MOF-808-EDTA-Cu. Red circles indicate the data points used for BET surface area calculations. The calculated BET areas for (c) MOF-808 and (d) MOF-808-EDTA-Cu, which were determined following the Rouquerol criteria 1-4 with BETSI program[2]. Not that, the calculated BET area of MOF-808 is 2065 m^2/g, which is consistent with previously reported values (1591~2424 m^2/g)[3-5]. Pore size distribution graphs of (e) MOF-808 and (f) MOF-808-EDTA-Cu.

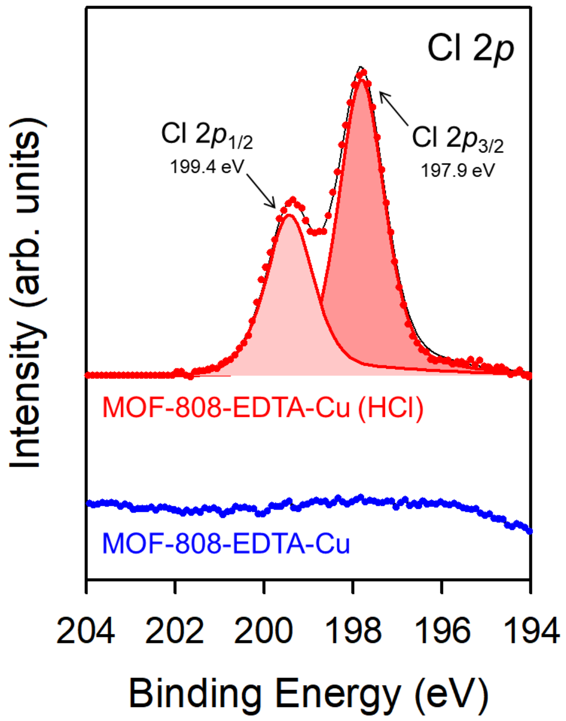

Supplementary Figure 9. Cl 2*p* XPS spectra of MOF-808-EDTA-Cu (HCl) (red dot) and MOF-808-EDTA-Cu (blue dot).

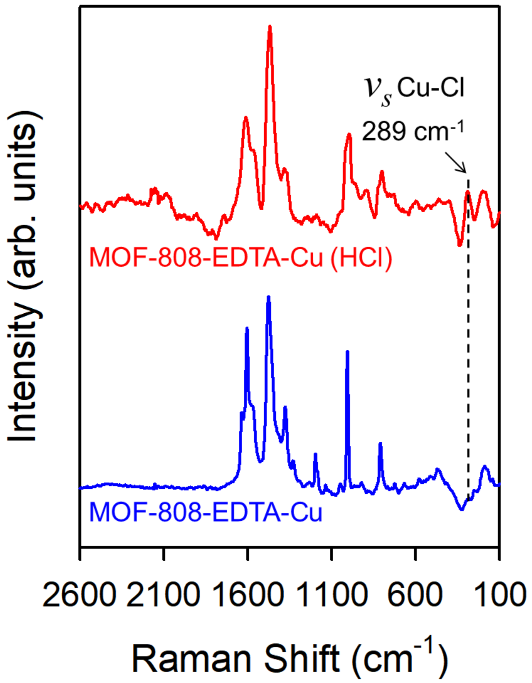

Supplementary Figure 10. Raman spectra of MOF-808-EDTA-Cu (HCl) (red) and MOF-808-EDTA-Cu (blue). For MOF-808-EDTA-Cu (HCl) (red), the peak appeared at 289 cm^{-1} is identical to the Raman stretching of Cu-Cl, which is formed with incorporated Cl to Cu^{2+} ion.

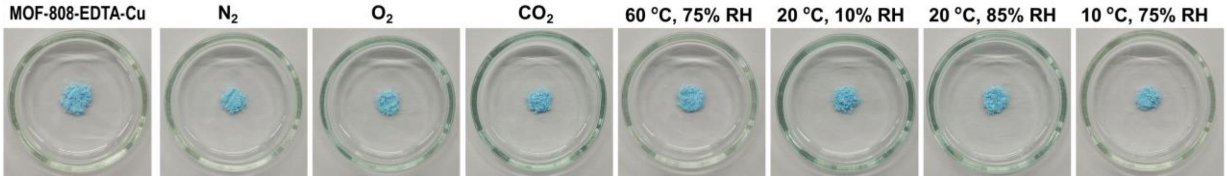

Supplementary Figure 11. Photographs of MOF-808-EDTA-Cu exposed to N_2, O_2, CO_2, 60 ℃, 75% relative humidity air, 20 ℃, 10% relative humidity air20 ℃, 85% relative humidity air, 10 ℃, 75% relative humidity air for 24 hours.

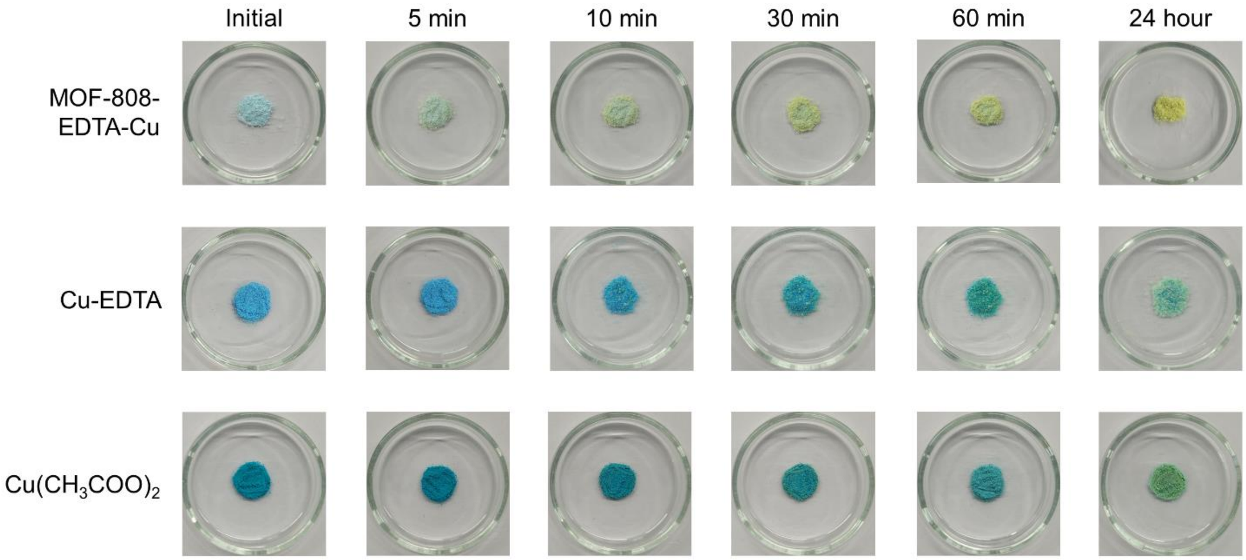

Supplementary Figure 12. Photographs of MOF-808-EDTA-Cu, Cu-EDTA, and $Cu(CH_3COO)_2$ for the colorimetric response by exposure to 120 ppm HCl vapor for 5 min, 10 min, 30 min, 60 min, and 24 hours.

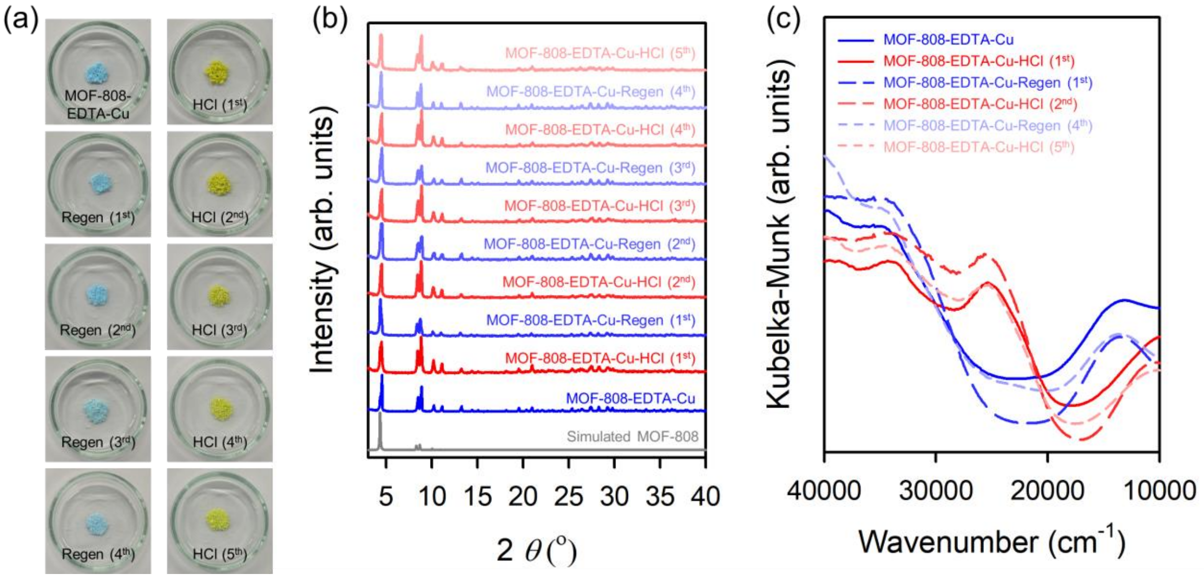

Supplementary Figure 13. (a) Photographs of MOF-808-EDTA-Cu during a series of hydrochloric acid vapor exposure and regeneration cycles. (b) XRPD patterns of MOF-808-EDTA-Cu at each stage during five cycles of alternating exposure to HCl vapor and water. (c) Diffuse reflectance UV-vis-NIR spectra of MOF-808-EDTA-Cu, 1st HCl exposed MOF-808-EDTA-Cu, 1st regenerated MOF-808-EDTA-Cu, 2nd HCl exposed MOF-808-EDTA-Cu, 4th regenerated MOF-808-EDTA-Cu, and 5th HCl exposed MOF-808-EDTA-Cu.

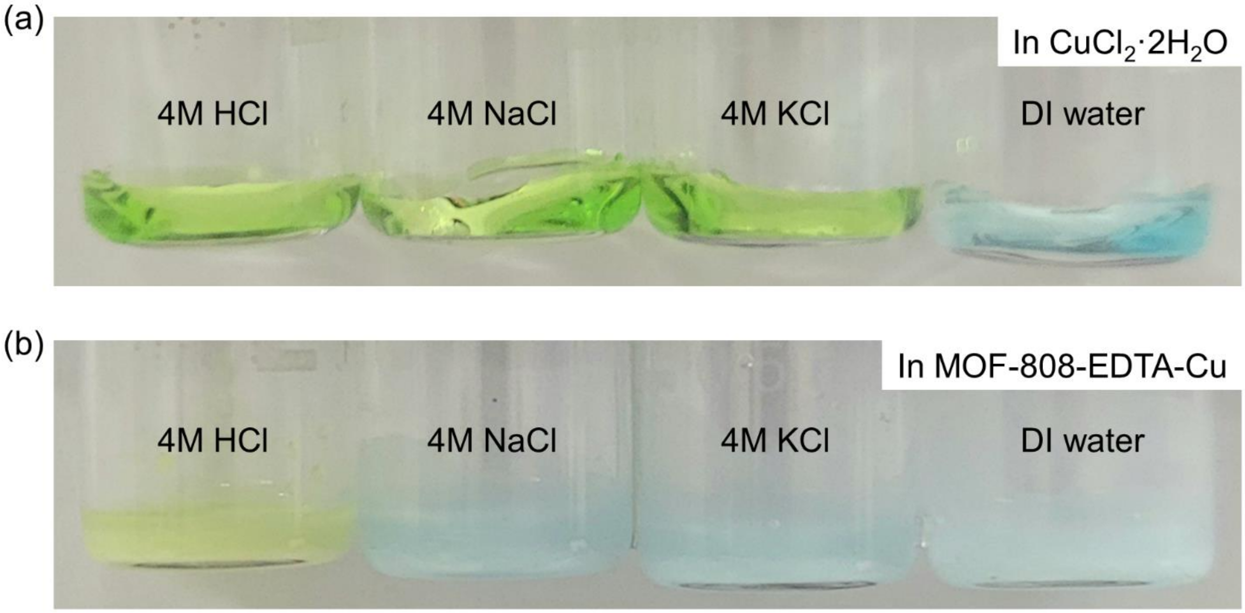

Supplementary Figure 14. Photographs of (a) $CuCl_2 \cdot 2H_2O$, (b) MOF-808-EDTA-Cu in 4M HCl, NaCl, KCl solution and DI water.

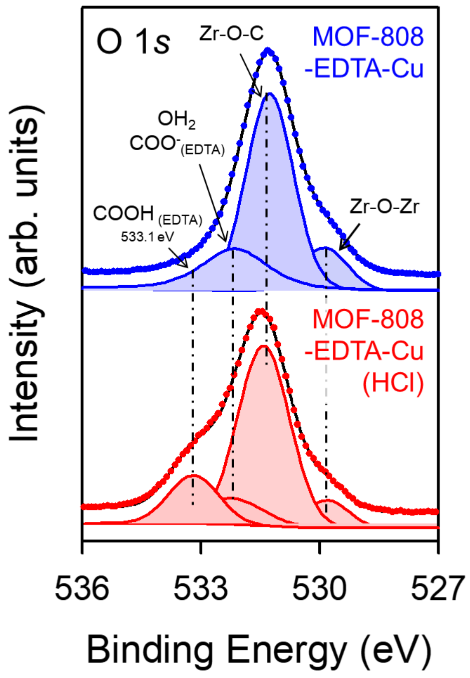

Supplementary Figure 15. O 1*s* XPS spectra of MOF-808-EDTA-Cu (blue) and MOF-808-EDTA-Cu (HCl) (red).

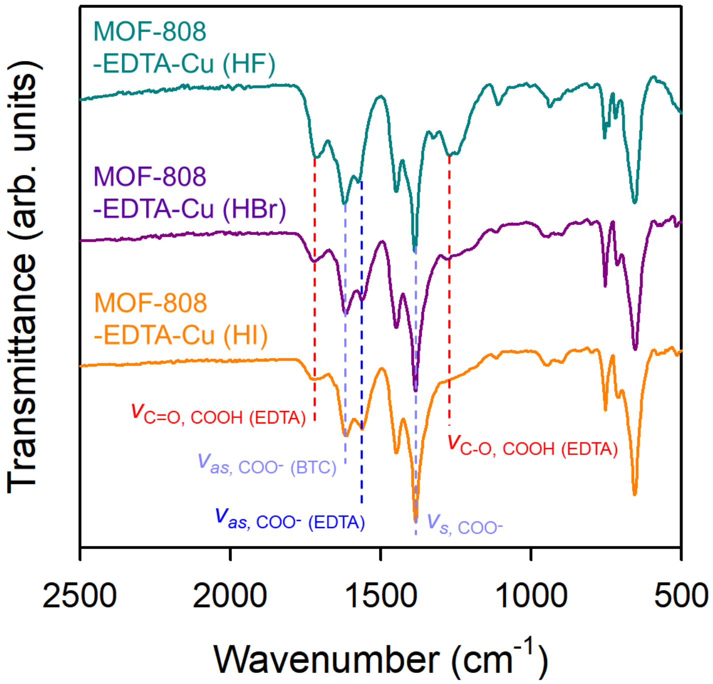

Supplementary Figure 16. FT-IR spectra of MOF-808-EDTA-Cu (HF) (dark green), MOF-808-EDTA-Cu (HBr) (dark purple) and MOF-808-EDTA-Cu (HI) (orange).

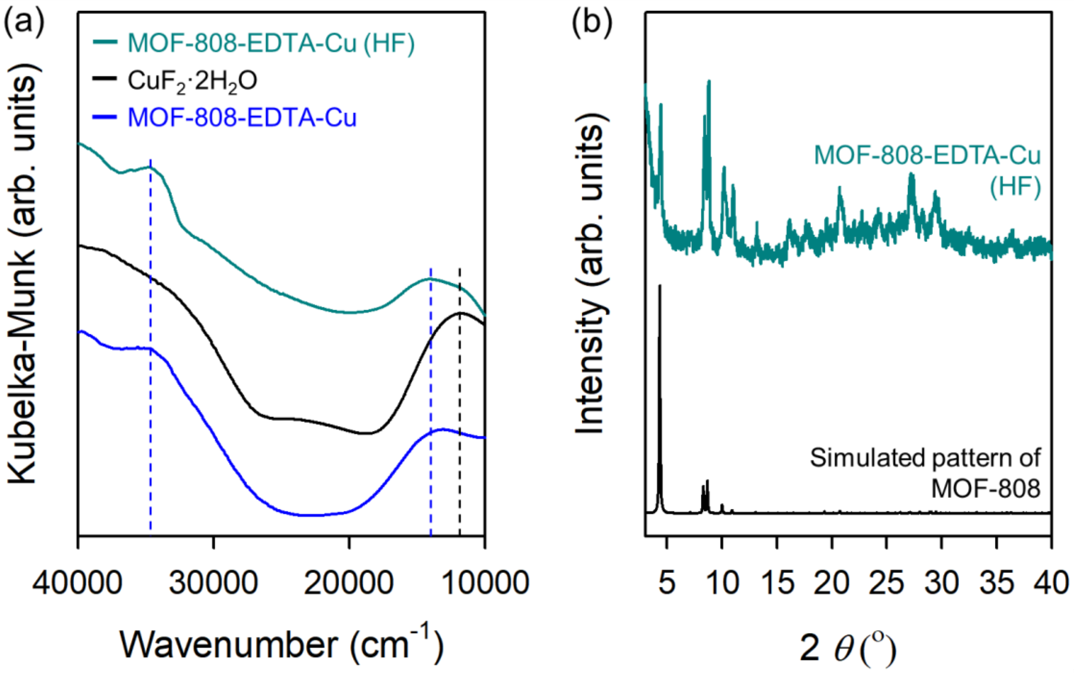

Supplementary Figure 17. (a) Diffuse reflectance UV-vis-NIR spectra of MOF-808-EDTA-Cu (HF) (dark green), $CuF_2 \cdot 2H_2O$ (black), and MOF-808-EDTA-Cu (blue). (b) XRPD patterns of MOF-808-EDTA-Cu (HF) (dark green) with simulated XRPD pattern of MOF-808 (black). Note that, high background intensity in XRPD pattern of MOF-808-EDTA-Cu (HF) implies that partial decomposition of MOF-808 structure.

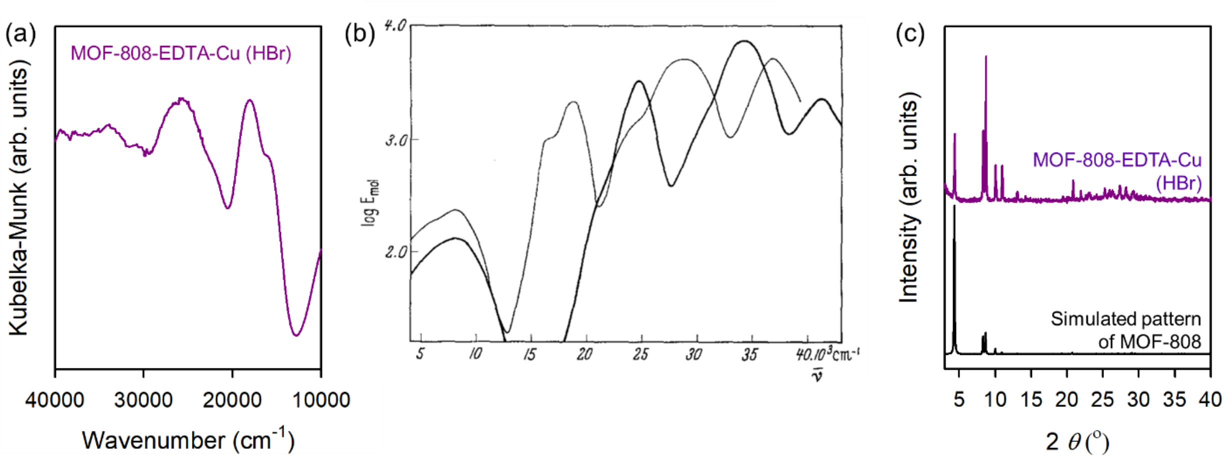

Supplementary Figure 18. (a) Diffuse reflectance UV-vis-NIR spectra of and MOF-808-EDTA-Cu (HBr), (b) Molar absorption spectra of $[CuCl_4]^{2-}$ (thick line) and $[CuBr_4]^{2-}$ (thin line). Reprinted with permission from ref 6. Copyright 1963 Springer Nature. (c) XRPD patterns MOF-808-EDTA-Cu (HBr) (dark purple) with simulated XRPD pattern of MOF-808 (black).

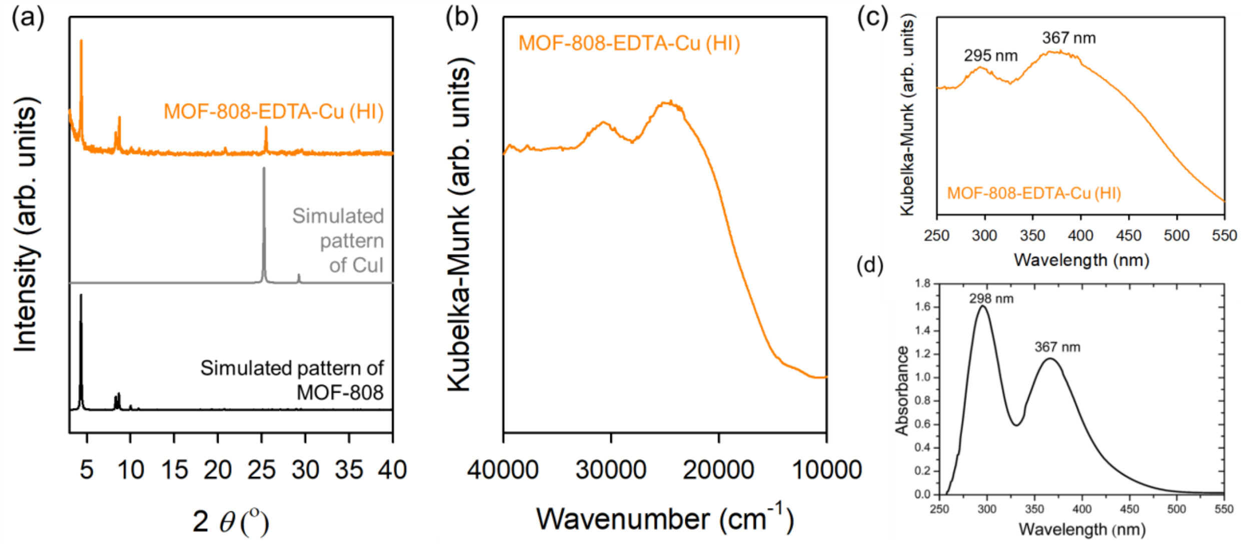

Supplementary Figure 19. (a) XRPD patterns of MOF-808-EDTA-Cu (HI) (orange) with simulated XRPD patterns of CuI (gray) and MOF-808 (black), (b) Diffuse reflectance UV-vis-NIR spectra of MOF-808-EDTA-Cu (HI) (orange). (c) Diffuse reflectance UV-vis-NIR spectra of MOF-808-EDTA-Cu (HI) in the 200-600 nm wavelength range. (d) Reported UV-vis spectrum of triiodide in ED-600/iodoethane/I_2 electrolyte showing absorption peaks of I_3^- at 298 and 367 nm. Reprinted with permission from ref 7. Copyright 2011 The Royal Society of Chemistry.

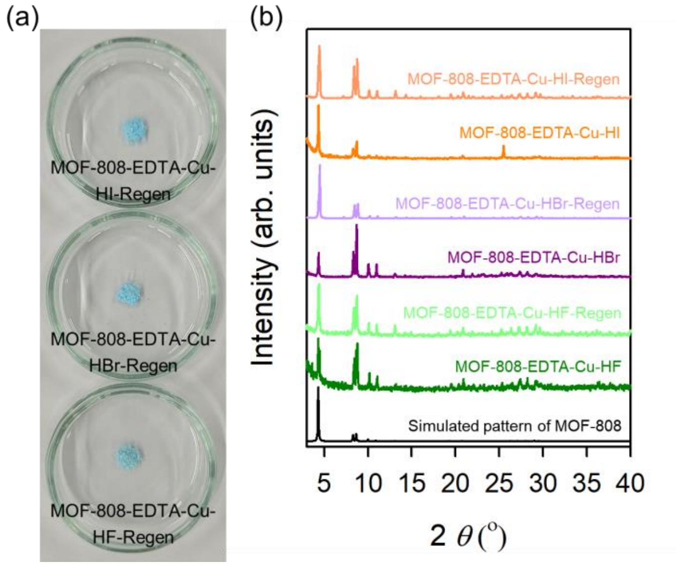

Supplementary Figure 20. (a) Photographs of MOF-808-EDTA-Cu-HF-Regen, MOF-808-EDTA-Cu-HBr-Regen and MOF-808-EDTA-Cu-HI-Regen. (b) XRPD patterns of MOF-808-EDTA-Cu-HI-Regen (light orange), MOF-808-EDTA-Cu-HI (orange), MOF-808-EDTA-Cu-HBr-Regen (light purple), MOF-808-EDTA-Cu-HBr (purple), MOF-808-EDTA-Cu-HF-Regen (light green) and MOF-808-EDTA-Cu-HF (green) with simulated XRPD patterns of MOF-808 (black).

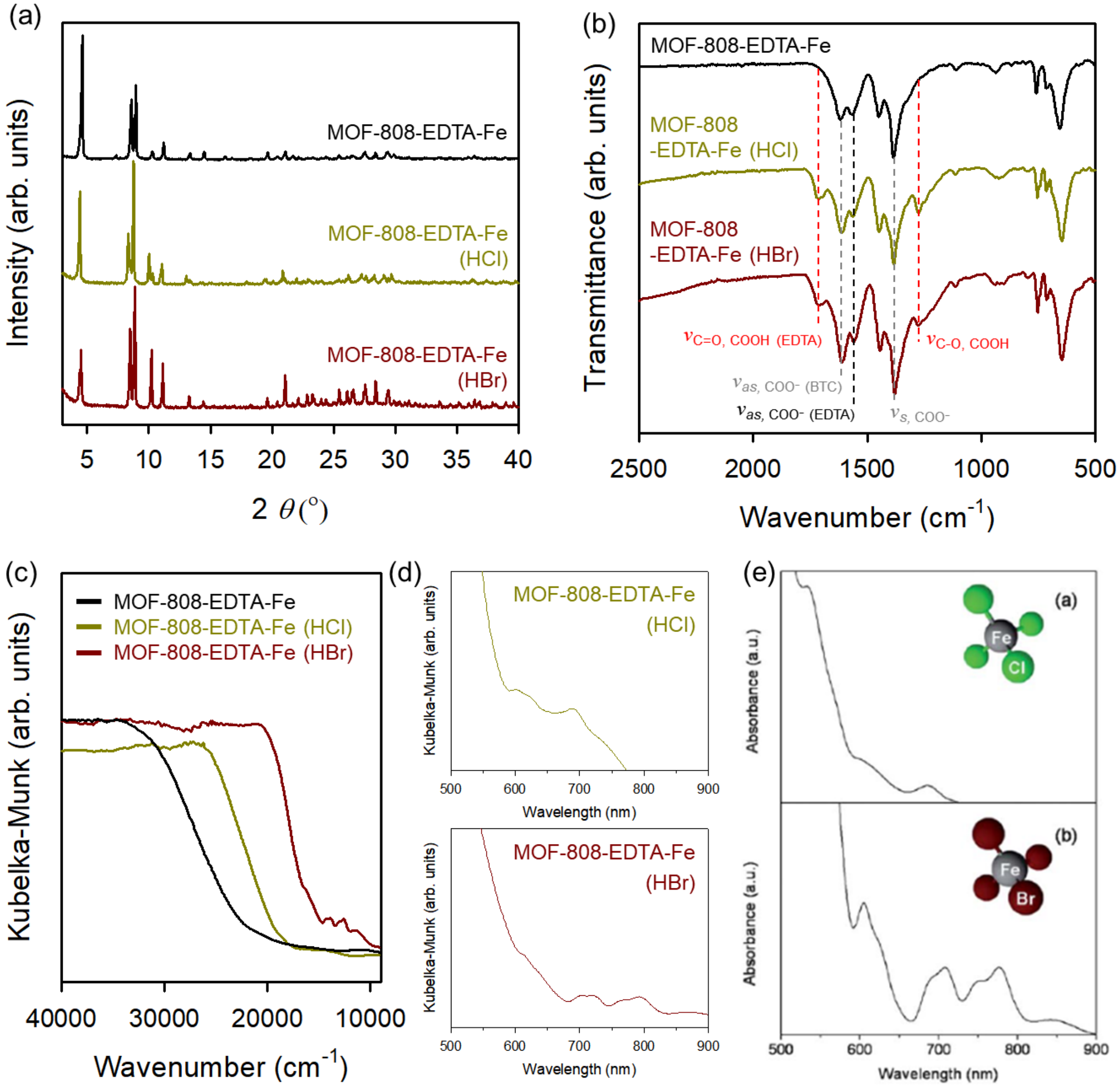

Supplementary Figure 21. (a) PXRD patterns of MOF-808-EDTA-Fe (black), MOF-808-EDTA-Fe (HCl) (dark yellow), and MOF-808-EDTA-Fe (HBr) (brown), (b) FT-IR spectra of MOF-808-EDTA-Fe (black), MOF-808-EDTA-Fe (HCl) (dark yellow), and MOF-808-EDTA-Fe (HBr) (brown). (c) Diffuse reflectance UV-vis-NIR spectra of MOF-808-EDTA-Fe (black), MOF-808-EDTA-Fe (HCl) (dark yellow), and MOF-808-EDTA-Fe (HBr) (brown). (d) Diffuse reflectance UV-vis-NIR spectra of MOF-808-EDTA-Fe (HCl) (dark yellow) and MOF-808-EDTA-Fe (HBr) (brown) in the 500-900 nm wavelength range. (e) Reported UV-vis spectra of $FeCl_4^-$ (upper) and $FeBr_4^-$ (down) in the 500-900 nm wavelength range. Reprinted with permission from ref 8. Copyright 2011 The Royal Society of Chemistry.

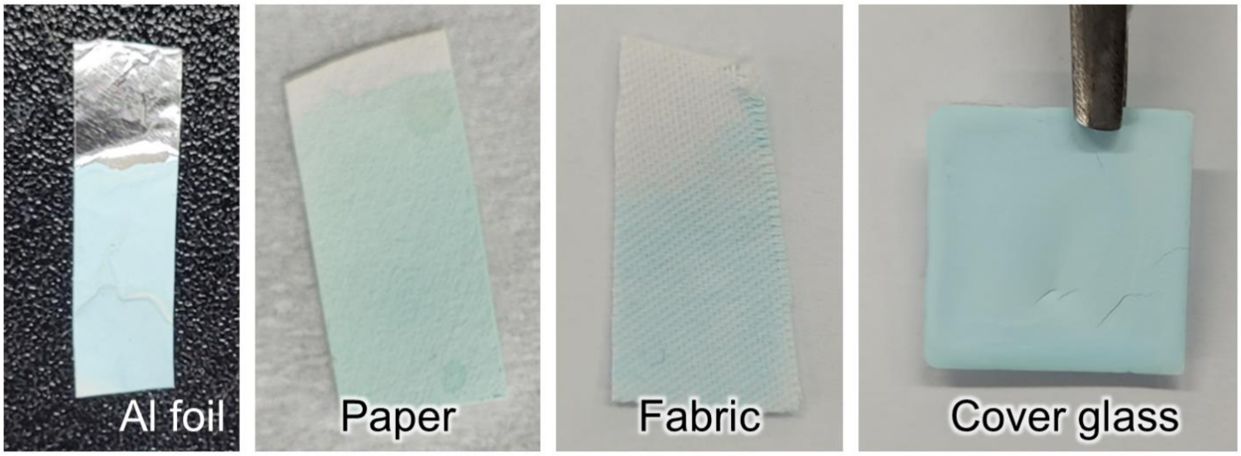

Supplementary Figure 22. Photographs of MOF-808-EDTA-Cu ink containing 20 wt% PVDF applied to various substrates.

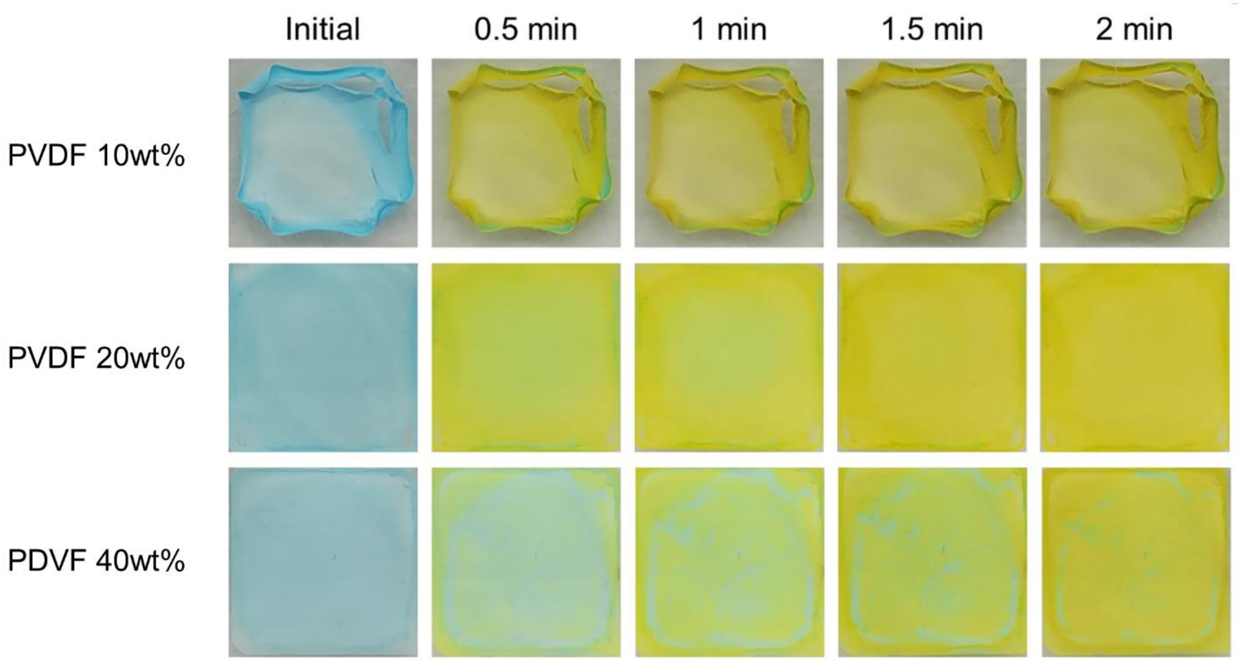

Supplementary Figure 23. Photographs of portable acid vapor decoding sensors processed on cover glass with varying PVDF amounts and after exposure to 15,500 ppm HCl vapor. The sensor with 20 wt% PVDF, tailored for optimal performance, demonstrated the best results, while A low PVDF mass ratio of 10 wt% resulted in poor film formation, and a high PVDF mass ratio of 40 wt% led to a diminished cyan-to-yellow transition within the same exposure timeframe.

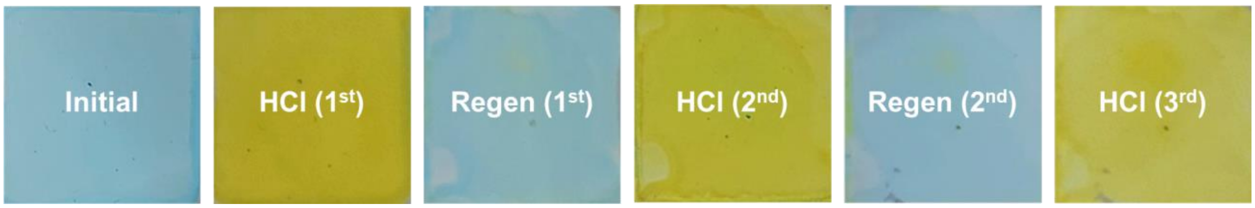

Supplementary Figure 24. Photographs of MOF-808-EDTA-Cu portable sensor with hydrochloric acid vapor exposure and regeneration series.

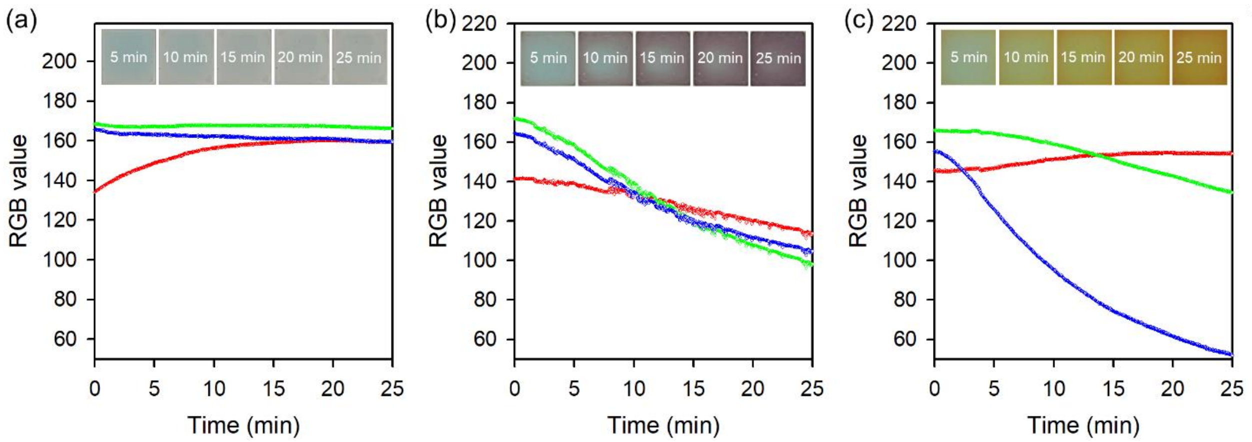

Supplementary Figure 25. Time-dependent RGB curves of MOF-808-EDTA-Cu portable sensors with 20 wt% PVDF exposed to (a) HF, (b) HBr, and (c) HI vapor. (Inset) Photographs of MOF-808-EDTA-Cu portable sensors with 20 wt% PVDF under acid vapor exposures.

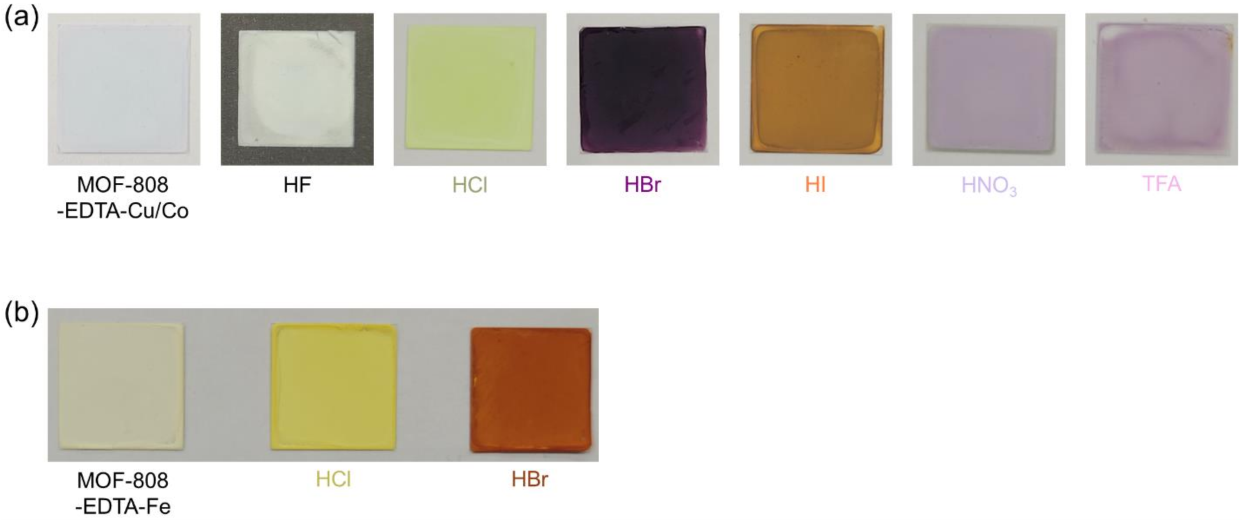

Supplementary Figure 26. (a) Color change of MOF-808-EDTA-Cu/Co portable sensor for identifying hydrofluoric acid, hydrochloric acid, hydrobromic acid, hydroiodic acid, nitric acid, and trifluoroacetic acid vapor, (b) Color change of MOF-808-EDTA-Fe portable sensor for identifying hydrochloric acid, and hydrobromic acid vapor.

Supplementary Table 1. Summary of representative acid vapor sensor studies.

Sensor	Sensor Mechanism	Acid Vapors	Ability to Decode	Ref.
MOF-808-EDTA-Metal	Proton-triggered metal-anion coordination	HCl, HF, HBr, HI, HNO_3, TFA	**Yes**	**This work**
**Organic dyes**				
1,3,3-Trimethylindolino-6’-nitrobenzopyrylospiran	Protonation	HCl	**No**	9,10
8-methoxy-1',3',3',-trimethyl-6-nitrospiro[chromene-2,2'-indoline]	Protonation	HCl	**No**	11
Sborepy3	Protonation	HCl	**No**	12
Sborepy6				
**Polymer**				
TATF-COM	Protonation	HCl	**No**	13
CC-TATP-COP	Protonation	HCl	**No**	14
**Molecular Metal Complexes**				
$[ZnHLCl_2]\cdot THF$	Phase change	HCl	**No**	15
$[ZnHLCl_2]\cdot DMF$				
$[Cd_2L_2Cl_4]\cdot CH_3CN$	Phase change	HCl	**No**	16
$ZnLCl_2$				
$CdHL_2Cl_2$	Protonation	HCl	**No**	17
**Covalent Organic Frameworks (COFs)**			**No**	
PhotoPAN	Protonation	HCl	**No**	18
Per-Py COF	Protonation	TFA	**No**	19
Per-N COF				
Py-TT COF				
CZ-DHZ-COF	Protonation	HCl	**No**	20
**Metal Organic Frameworks (MOFs)**				
$Zn_{14}(TCPE)_5(NO_2)_4(O)_6$<br>$Co_{14}(TCPE)_5(NO_2)_4(O)_6$	HCl adsorption, dipole effect	HCl	**No**	21
CSMCRI-6	Protonation	HCl	**No**	22
**Colorimetric Sensor Arrays**				
36 different responsive pigments<br>(Methyl Red, Fluorescein, Reichart’s Dye, $Pb(OAc)_2$, etc.)		HF, HCl, HNO_3	**Yes**	23

Supplementary Table 2. ICP-AES analysis results for MOF-808-Cu, MOF-808-EDTA-Cu, and MOF-808-EDTA-Cu-regen.

	Zr		Cu		Zr : Cu (molar ratio)
	mg/L	mmol/L	mg/L	mmol/L	
MOF-808-EDTA-Cu	78.457	0.860	13.193	0.208	1.000 : 0.242
MOF-808-EDTA-Cu-Regen (1st)	17.093	0.187	2.265	0.036	1.000 : 0.193
MOF-808-EDTA-Cu-Regen (2nd)	21.414	0.235	2.185	0.034	1.000 : 0.145
MOF-808-EDTA-Cu-Regen (3rd)	21.163	0.232	1.873	0.029	1.000 : 0.125
MOF-808-EDTA-Cu-Regen (4th)	13.761	0.151	0.650	0.010	1.000 : 0.066
MOF-808-Cu [a]	84.745	0.929	0.079	0.001	1.000 : 0.001

[a] MOF-808 (without EDTA) was immersed in 10 mL of a 0.1 M aqueous Cu^{2+} solution for 24 hours. ICP-AES analysis showed a nearly negligible amount of Cu^{2+} in MOF-808-Cu, indicating that the majority of Cu^{2+} present in MOF-808-EDTA-Cu is due to the chelation effect of EDTA.

Supplementary Table 3. Calculation of the percentage of Cu^{2+} chelated by EDTA in MOF-808-EDTA-Cu.

	EDTA : BTC[a,b]	BTC[a] : Zr[c]	Zr : Cu[d]	EDTA : Cu
MOF-808-EDTA-Cu	0.89 : 1.00	1.00 : 3.00	3.00 : 0.72	0.89 : 0.72 (82%)

[a] Abbreviation for 1,3,5-benzenetricarboxylic acid
[b] The ratio of EDTA to BTC confirmed by NMR results (Supplementary Fig. 2)
[c] The ratio of BTC to Zr in MOF-808 ($Zr_6O_4(OH)_4(BTC)_2(HCOO)_6$)
[d] The ratio of Zr to Cu determined by ICP-AES results (Supplementary Table 1)

References for supplementary information

1. Yu, K. et. al. A low-cost commercial Cu (ii)–EDTA complex for electrocatalytic water oxidation in neutral aqueous solution. *Chem. Commun.* **58**, 12835-12838 (2022).

2. Osterrieth, J. W. et al. How reproducible are surface areas calculated from the BET equation?. *Adv. Mater.* **34**, 2201502 (2022).

3. Furukawa, H. et al. Water adsorption in porous metal–organic frameworks and related materials. *J. Am. Chem. Soc.* **136**, 4369-4381 (2014).

4. Peng, Y.et al. A versatile MOF-based trap for heavy metal ion capture and dispersion. *Nat. Commun.* **9**, 187-195 (2018).

5. Aunan, E. et al. Modulation of the Thermochemical Stability and Adsorptive Properties of MOF-808 by the Selection of Non-structural Ligands. *Chem. Mater.* **33**, 1471-1476 (2021).

6. Furlani, C. & Morpurgo, G. Properties and electronic structure of tetrahalogenocuprate (II)-complexes. *Theor. Chim. Acta* **1**, 102-115 (1963).

7. Apostolopoulou, A., Margalias, A. & Stathatos, E. Functional quasi-solid-state electrolytes for dye sensitized solar cells prepared by amine alkylation reactions. RSC Adv. 5, 58307-58315 (2015).

8. Döbbelin, M. et al. Synthesis of paramagnetic polymers using ionic liquid chemistry. *Polym. Chem.* **2**, 1275-1278 (2011).

9. Nam, Y. S. et al. Photochromic spiropyran-embedded PDMS for highly sensitive and tunable optochemical gas sensing. *Chem. Commun.* **50**, 4251-4254 (2014).

10. Guo, J., Wei, X., Fang, X., Shan, R. & Zhang, X. A rapid acid vapor detector based on spiropyran-polymer composite. *Sens. Actuators B Chem.* **347**, 130623 (2021).

11. Genovese, M. E. et al. Light responsive silk nanofibers: an optochemical platform for environmental applications. *ACS Appl. Mater. Interfaces* **9**, 40707-40715 (2017).

12. Li, K. et al. Solvatochromism, acidochromism and aggregation-induced emission of propeller-shaper spiroborates. *Dalton Trans.* **47**, 15002-15008 (2018).

13. Subodh, Prakash, K. & Masram, D. T. Chromogenic covalent organic polymer-based microspheres as solid-state gas sensor. *J. Mater. Chem. C.* **8**, 9201-9204 (2020).

14. Subodh, Prakash, K. & Masram, D. T. A reversible chromogenic covalent organic polymer for gas sensing applications. *Dalton Trans.* **49**, 1007-1010 (2020)

15. Liang, Q.-F., Zheng, H.-W., Yang D.-D. & Zheng, X.-J. Zn(II) complexes based on a Schiff base: mechanochromism- and solvent molecule-dependent acidochromism. *Cryst. Growth Des.* **22**, 3924-3931 (2022).

16. Yang, D.-D. et al., Multi-stimuli responsive behavior of two Schiff base complexes with high contrast multicolor switching and wearable applications for rapid detection of HCl and NH3 vapor. *Dyes and Pigments* **212**, 111149 (2023).

17. Liang, Q.-F., Zheng, H.-W., Yang, D.-D. & Zheng, X.-J. A triphenylamine derivative and its Cd(II) complex with high-contrast mechanochromic luminescence and vapochromism. *CrystEngComm* **24**, 543-551 (2022).

18. Kundu, P. K., Olsen, G. L., Kiss, V. & Klajn, R. Nanoporous frameworks exhibiting multiple stimuli responsiveness. *Nat. Commun.* **5**, 3588 (2014)

19. Ascherl, L. et al. Perylene-based covalent organic frameworks for acid vapor sensing. *J. Am. Chem. Soc.* **141**, 15693-15699 (2019).

20. Gong, W. et al. Dual-function fluorescent hydrazone-linked covalent organic frameworks for acid vapor sensing and iron (iii) ion sensing. *J. Mater. Chem. C* **10**, 3553-3559 (2022).

21. Zhu, Z. H., Ni, Z., Zou, H. H., Feng, G. & Tang, B. Z. Smart metal-organic frameworks with reversible luminescence/magnetic switch behavior for HCl vapor detection. *Adv. Func. Mater.* **31**, 2106925 (2021).

22. Goswami, R., Das, S., Seal, N., Pathak, B. & Neogi, S. High-performance water harvester framework for triphasic and synchronous detection of assorted organotoxins with site-memory-reliant security encryption via pH-triggered fluoroswitching. *ACS Appl. Mater. Interfaces* **13**, 34012-34026. (2021).

23. Feng, L. et al. Colorimetric sensor array for determination and identification of toxic industrial chemicals. *Anal. Chem.* **82**, 9433-9440 (2010).
