# Supplementary material for: Colorimetric identification of colorless acid vapors using a metal-organic framework-based sensor
Source: Nat Commun. 2025 Jan 4;16:385. doi: 10.1038/s41467-024-55774-x (PMC11700211; doi:10.1038/s41467-024-55774-x)
Supplement: Supplementary file 2 — Description of Additional Supplementary Files [file 41467_2024_55774_MOESM2_ESM.pdf]

Description of Additional Supplementary Files

Supplementary Movie 1

Description: HCl vapor detection of MOF-808-EDTA-Cu. A 3 cm glass dish containing 0.010 g of MOF-808-EDTA-Cu was placed inside a 5 cm glass dish with 2 mL of concentrated HCl solution, ensuring no direct contact between the MOF-808-EDTA-Cu and the acid solution. The 5 cm dish was covered with a 7 cm glass dish to detect vaporized acid. The numbers at the bottom of the video indicate the reaction time.
